# Inteins in Microbial Genomes: Distribution, Mechanism, and Function

**DOI:** 10.1100/tsw.2002.13

**Published:** 2002-05-29

**Authors:** Maurice W. Southworth, Francine B. Perler

**Affiliations:** New England Biolabs, 32 Tozer Road, Beverly, MA 01915, UK

## Abstract

Inteins are self-splicing protein elements (134 to 608 amino acids). Over 125 inteins have been cataloged in InBase, the on-line intein database (http://www.neb.com/neb/inteins.html), which includes the Intein Registry[1]. Inteins naturally present in pathogenic microbes represent novel, yet unexploited drug targets. Understanding the chemistry of the splicing reaction has allowed the manipulation of inteins, which are now used in many protein engineering applications[2].

**RESULTS AND DISCUSSION**. Most inteins have been discovered during microbial genome sequencing projects. Identification of new inteins from sequenced genomes is facilitated by the presence of signature motifs[1]. Functional genomic tools translate DNA sequence information into structure and function. Similarly, the discovery of new intein DNA sequences brings information about essential enzyme motifs, since inteins are usually inserted in active sites or binding pockets of enzymes. Moreover, inteins tend to be found in functionally important or ancient host proteins. 34 different proteins contain 1-3 inteins at 52 different insertion sites. 19 intein-containing proteins are involved in DNA repair, replication, and gene expression, while 15 are not. 50 organisms from 38 genera contain inteins. The large number of inteins found in some species, especially archaea, may be indicative of a higher rate of genetic exchange, possibly an advantage to organisms living in extreme environments.

Larger inteins contain homing endonuclease domains that can initiate intein gene mobility. Thus, inteins can be inherited vertically or horizontally. Although no intein has been found in a multicellular organism, the hedgehog signaling protein, which is required for pattern formation during embryogenesis, is derived from a precursor containing an intein-like autoprocessing domain[3]. Inteins may have played an early role in evolution by mediating domain shuffling prior to the development of nucleic acid recombination or by allowing one domain to separately pair with multiple domains.

The self-catalytic protein splicing mechanism requires the intein plus the first C-terminal extein (host protein) residue. Biochemical, genetic, and biophysical approaches have been used to define the protein splicing mechanism[4]. Although inteins are not classic catalysts or enzymes since they do not act on multiple substrates, they use similar strategies to cleave splice junctions and ligate exteins. A single intein active–site directs 3 distinct, but coordinated, nucleophilic displacements. Polymorphisms in nucleophiles and conserved residues have become more evident as more inteins have been sequenced. A second protein splicing mechanism has been determined for inteins lacking an N-terminal nucleophile[5].

InBase[1] is a curated database providing general information on protein splicing as well as detailed information on mechanism, conserved sequences, insertion sites, and intein properties. InBase also contains contact information and an annotated bibliography with PMID links. An intein-specific BLAST server allows on-line identification of inteins in query sequences.
